# Implementation of the Haptic Tele-Weight Device Using a 10 MHz Smart Torch VLC Link

**DOI:** 10.3390/mi13112031

**Published:** 2022-11-20

**Authors:** Aqeel Farooq, Xiping Wu

**Affiliations:** School of Electrical and Electronic Engineering, University College Dublin, Belfield, D04 V1W8 Dublin, Ireland

**Keywords:** 6G, VLC, e-commerce, HX711, GUI, LED, pre-equalizer, post-equalizer, tele-weight, haptic interaction, embedded, IoT

## Abstract

Considering the prerequisite need for a protected e-commerce platform, absence of haptic interaction in head-mounted displays (HMD), and exploitation of faster communication technology, this research work aims to present an amended version of the tele-weight device that utilizes the 6G visible light communication (VLC) technology, is faster in performance, and deals with a heavier article. The enhanced version of the device is to be called the ‘VLC tele-weight device’ and the aim for the VLC tele-weight device is to get it affixed over the headset which will allow the user to have the weight-based sensation of the product ordered on the virtual store. The proposed device sending end and receiving end part performs communication over the VLC link. Furthermore, Arduino Nano is used as the microcontroller (MCU) in the project. Sending end circuitry measures the weight using the load cell and HX711 amplifier combination and transmits it via the connected LED. The pre-equalizer circuit is connected between the LED and sending end part to improve the bandwidth. On the receiver side, the post-equalizer circuit improves the shape of the received pulse. The received weight value is then displayed using the motor-gear combination. The sending end device is to be sited at the virtual store, while the receiving end is planned to be positioned over the VR headset. The performance of the device was measured by performing repeated trials and the percentage error was found to be between 0.5–3%. Merging the field of embedded systems, internet of things (IoT), VLC, signal processing, virtual reality (VR), e-commerce, and haptic sensing, the idea proposed in this research work can help introduce the haptic interaction, and sensational realization-based innovation in immersive visualization (IV) and graphical user interface (GUI) domain.

## 1. Introduction

Haptic interaction (HI) devices or human-computer interface (HCI) machines include a system accumulation by the amalgamation of sensors, actuators, embedded system hardware, control system (feedback topology), and communication system technology, thereby allowing interaction of the human body involving movements and sensation with a computer. Technologies including VR, augmented reality (AR), mixed reality (MR), and visible interactive displays (VIDs) (headsets such as Oculus Meta Quest, HTC Vive, Lenovo Think Reality, etc.), are used to demonstrate and visualize graphics [1,2]. One of the advantages of having the VIDs is to have MR and VR-built virtual stores (VS) that allow a user to graphically visualize the product; however, due to the unavailability of quality haptic sensing technology (HST), it is not possible to physically interact with the product. Using the VLC link, this research work intends to propose and present an upgraded version of the tele-weight device created by Farooq et al. [3].

In optical wireless communication (OWC), VLC systems are tempting and appealing due to the utilization of the light-emitting diodes (LED) already available and being used in commercial applications ranging from mobile phones and lighting systems to TV panels and displays [4]. VLC is effective because a source generating light can also fulfill the purpose of transmitting data; however, a renowned bottleneck in the VLC technology is the slow response and low bandwidth of the commercial LEDs. The bandwidth of a commercial LED is increased using pre-equalization technology and blue filtering [5,6]. Using VLC communication, data in the form of light are transmitted at a blinking rate (BR) higher than perception sensitivity [7,8]. To increase the bandwidth of the LED at the transmission side, pre-equalization is practiced by means of the hardware circuit termed the analog pre-equalization (APE) and using software algorithms programmed on FPGAs or DSP boards labeled as digital pre-equalization (DPE) [9]. As APE allows the hardware level flexibility and therefore, to have more control over the components used, it is utilized in this project. With the aid of pre-equalization technology, the 3 dB bandwidth of the commercially available phosphor LED can be extended to 25 MHz from 1 MHz [10]. For this research work, a bandwidth of around 10 MHz is achieved using the hardware pre-equalizer circuit.

Different VLC links integrating organic LEDs or high brightness (HB-LEDs) for sending modulated data as optical transmitters have been created by our group and many others with data rates exceeding over 10 Mb/s with commercial LED (having a bandwidth of around 1 MHz) utilizing both APE as well as DPE and post-equalization technology [11]. In VLC, the information is transmitted by modulating the intensity of light waveform *s(t)*. The waveform *s(t)* is emitted by HB-LEDs and is demodulated at the receiver side using a photodetector, thereby, referring to the technique in which optical power is modulated to be direct detection (DD)/intensity modulation (IM). With the combination of systems jointly formed using photo-diod4ees and LEDs, sensor data can be transmitted over the line of sight (LOS) and non-LOS conditions. Data from the sensing technological devices can be sent over the channel, using the transmitter, received by the optical receiver [12,13].

The objective of this research work is to present a VLC technology tele-weight device using the concept of a *“smart torch”* whose resolution is to transmit the weight sensor data to the receiver torch-based device. The investigation focuses on displaying the weight of the item together with the VR headset focusing on introducing haptic interaction. Once the user picks an item in the store, the corresponding weight value will be transmitted between the two links and the IoT collaboration allows the user to feel the item weight in Dublin, Ireland while the store end of the device is Lahore, Pakistan. The performance evaluation containing parameters such as confusion matrix and accuracy of the VLC tele-weight device is computed.

## 2. Related Work/Literature Review

### 2.1. Evolution from 5G and 6G Framework

The introduction of the fifth generation (5G) communication technology led to the debate concerning whether 5G is a ‘revolution’ or ‘evolution’ compared to the prior legacy communication technologies but as the 5G is commercially available, the direction of the researcher’s focus has recently shifted towards the newly incoming sixth generation (6G) [14,15]. According to Tariq et al. [16], the 6G technology is expected to be released by 2028. The authors further emphasized that a sum-up of 11 technologies are a part of the 6G network including tetra hertz (THz) communication, multiple access points, VLC, channel coding, intelligent reflecting surfaces (IRS), artificial intelligence (AI), machine learning (ML) optimized algorithms, and zero energy massive multiple-input multiple-output (m-MIMO) interfaces [17,18]. Song et al. [19] state that VLC technology, as a part of the 6G technology, has excellent potential in transmitting data for short and medium-range communication technologies.

**Figure 1:**
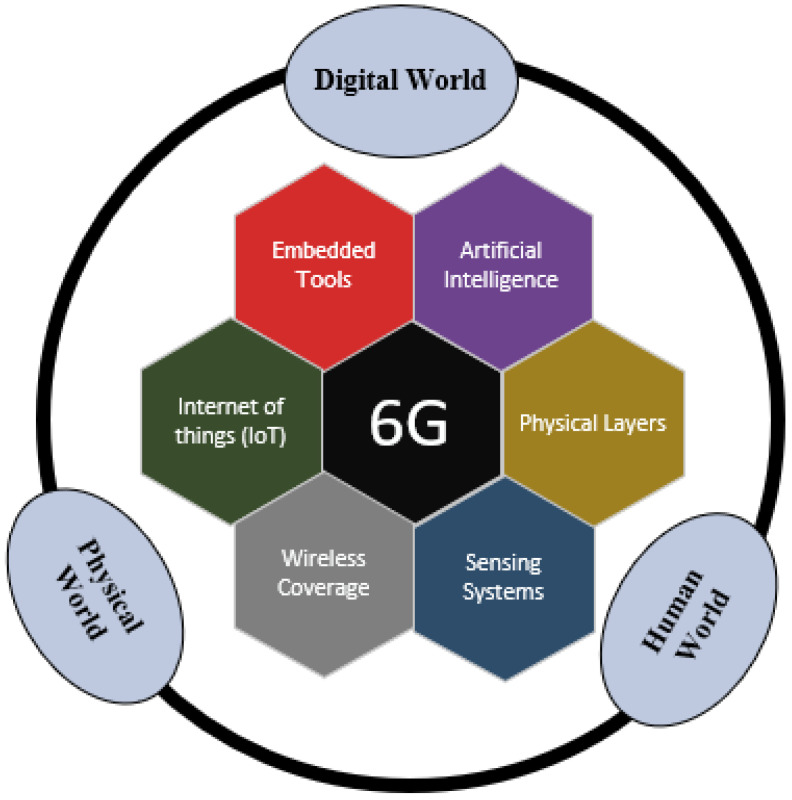
The possible framework of 6G [21].

Viswanathan and Mogensen [20] in their work stated that the 6G technological devices will be based on the embedded system hardware and smart algorithms compared to the traditional devices and will contain wearable touchscreen interfaces linking further technological features such as self-navigations, digital cash, domestic problem-solving skills, and health care aid. Imoize et al. [21] propose that improvements in enabling technologies such as blockchain, IRS, terahertz communication, smart algorithms, nano-electronics, smart energy materials, and energy harvesting techniques are necessary for the coverage and performance improvements in 6G communication (framework as illustrated in Figure 1).

### 2.2. Embedded System, IoT, and Proprioceptive Haptic Sensing Interfaces

Rodríguez et al. [22] revealed that in order to meet the need for adaptive edge computing in cyber-physical systems (CPS), a performance-embedded system is required with a custom electronics design to achieve optimal performance. According to Ashjaei et al. [23] with regard to the mechanical industry-driven focus on automated and self-driven cars, embedded software, IoT, and smart algorithms have been proven to be key enablers for attaining features and functionality in automotive systems relating to mining vehicles, construction bots, recycling machines, cranes and other off-road robots. With IoT, the data from the embedded hardware can be visualized over the network. Thangavel and Shokkalingam [24] in their work created an IoT-based system that detects animals and avoids human conflict with wildlife. The system proposed is adaptable in the zoo environment to avoid fatal wild–human life conflict. Zhou and Nie [25] propose that with the inclusion of the IoT, decision-making and fault detection capability over the embedded system network is improved (especially in medical diagnostics).

Rossi et al. [26] introduced the term “*HapPro*” for proprioceptive haptic interfacing and suggest a wearable pattern for feedback sensing (proprioceptive). Yin et al. [27] research work introduced an expanded thumb haptic interface using magnetorheological fluids. Garcia et al. [28] created a simulation that runs with the haptic interface-based button. Further, in their work, they propose a click zone area, interacting which makes the simulation runs.

### 2.3. VLC Equalization

Rajbhandari et al. [29] states that IEEE 802.15.7 is an eminent standard that proposes the solution to the problem caused by ambient sources, leading to the variation in the bit error rate (BER) and bit rate (BR) for short-range communication; moreover, recent advancements in the efficiency of the LEDs have made it possible for the VLC technology to replace them with the conventional RF systems.

Recently being a part of the 6G, VLC technology has drawn attention as a high-speed connectivity solution and following the Kisacik et al. [30] work, it can replicate the normal LED transmission of a signal of around or less than 1 MHz, therefore, a pre-equalization circuit is required to increase the bandwidth. After increasing the bandwidth once the signal is transmitted, post-equalization is practiced by deploying either the DPE or APE, to improve the signal at the receiver side, and one of the examples exploiting digital pre-equalizer with post-equalizer is created in Chen et al. [31] work.

## 3. Methodology

### 3.1. Working Principle of the Device

**Figure 2:**
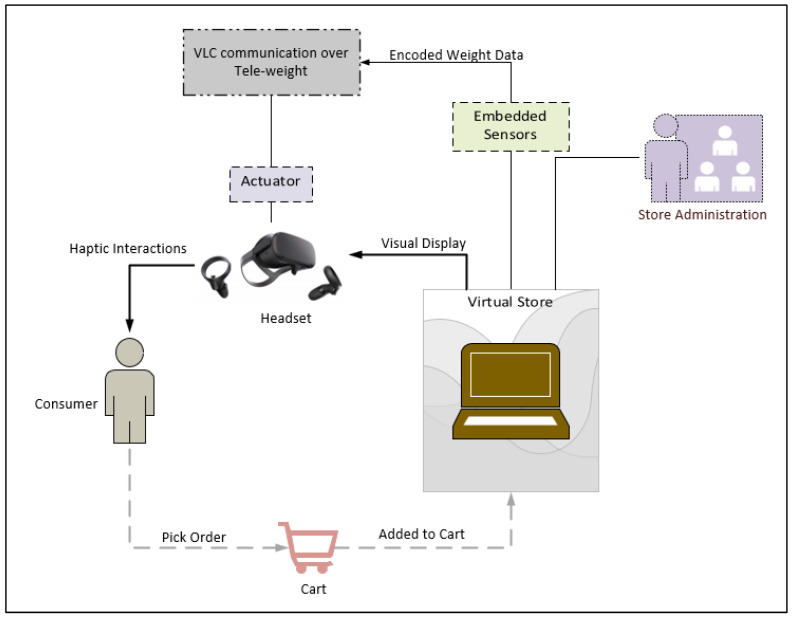
VLC Tele-weight Conceptual diagram.

The original purpose and goal of the VLC tele-weight device are to introduce improvement in the e-commerce shopping sector by reducing fraudulent activities (conceptual diagram is shown in Figure 2). The inception for the device is to get it fixed over the HMD display and roll the weight on the user’s hand depending on the net equivalent force to be shown. The working principle of the device includes the circuitry that makes the motor rotates. Once the user picks an item from the VR store (sending end), the corresponding sensors detect the corresponding weight and transmit data over the VLC link.

The VLC link at the receiver side makes the motor rotate (receiving end) accordingly, to show the weight of the item corresponding to the cart item. The HMD allows for the visualization, while the VLC tele-weight device introduces the haptic interaction with the product from the store. With the pre-equalization technology, the VLC link can send a signal at the frequency of 10 MHz which is faster than the previously created tele-weight device.

### 3.2. Hardware Design for Performance Evaluation

**Figure 3:**
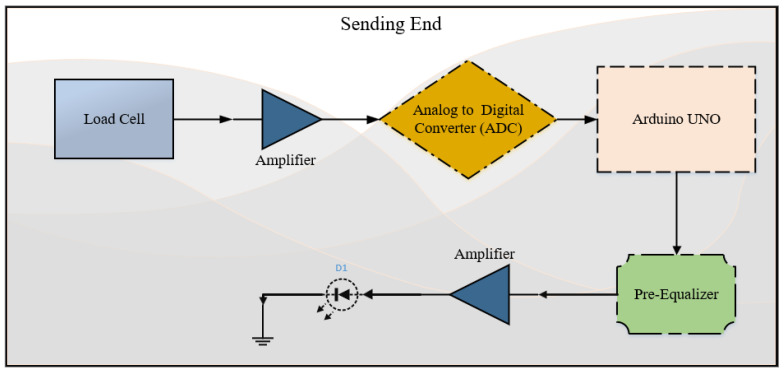
Proposed VLC tele-weight Sending End.

The concept for the VLC haptic tele-weight device created in this research is split into two sections. One part is regarded as the sending end and the other half is termed the receiving end. The VLC communication link composed of the pre-equalizer hardware, phototransistor, and transmitter LED is also of significant importance in the proposed configuration. The sending end circuitry (Figure 3) consists of the Arduino Nano device whose firmware is coded to collect the amplified signal from the weight sensor (load cell). The load cell voltage signal is smaller; therefore, it needs amplification before meaning information is obtained from the generated voltage value. From the Arduino, the corresponding PWM is generated at 7–10 MHz and the pre-equalizer circuit allows the commercial LED to transmit a high-frequency signal. The number of pulses to be sent is based on the weight (three-digit accuracy, 5.82 kg corresponds to the 582 pulses). The overall device is designed for the capability of a 10 kg configuration.

**Figure 4:**
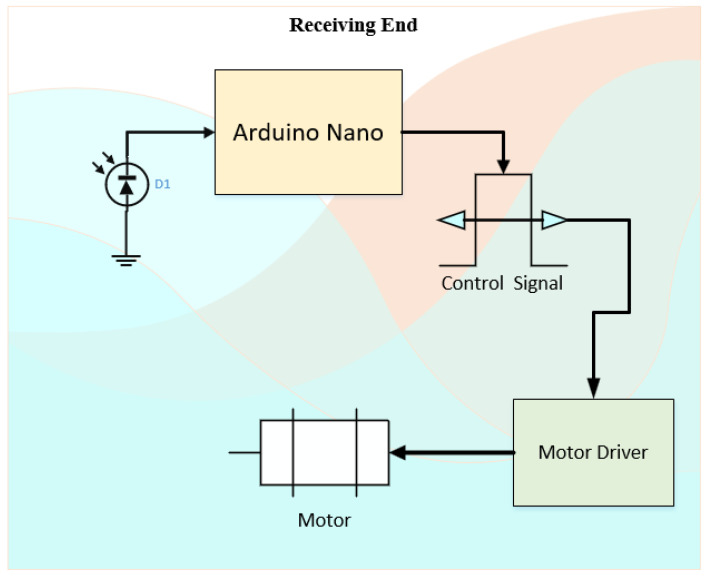
Proposed VLC tele-weight receiving end.

The receiving end (in Figure 4) also uses the same Arduino Nano device. The connected phototransistor in combination with the resistor allows for the reception of the light signal. With the determination of the light pulses, the pulse signal for the motor is generated based on the current position of the motor. To determine the performance of a device, a confusion matrix is created to obtain accuracy-related information.

**Figure 5:**
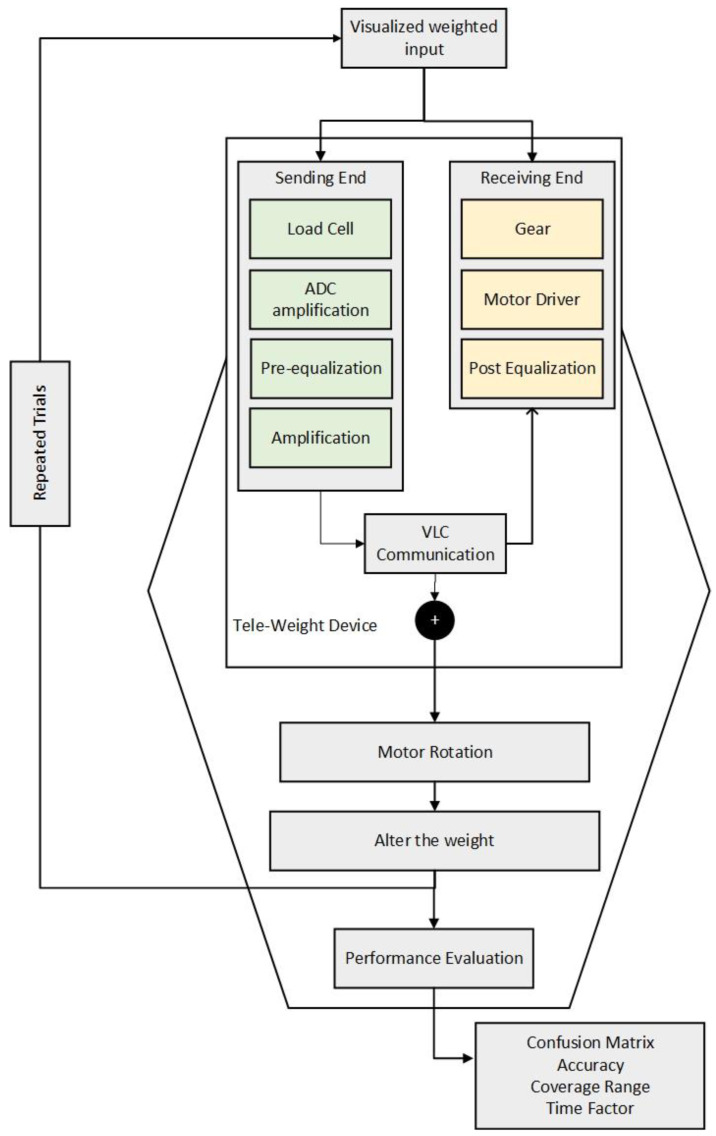
Block diagram for performance evaluation approach.

Figure 5 demonstrates the mechanism planned for the determination of the performance evaluation. The device capability with the previous version is 5 kg which is now increased to 10 kg with a more capable load cell. The advantage of haptically realizing the weight is to have an IV experience together with the in-hand experience (feeling) of the item. At first, the prototype is created and then repeated trials are performed to determine the relevant information about the accuracy, confusion matrix, and other relevant parameters.

## 4. Results

In this research work, a VLC tele-weight device is proposed which works by transmitting data over the 6G VLC technology (Li-Fi, light fidelity). Introducing innovation in the e-commerce sector, the key objective of this device is to allow the customer to realize and feel the object’s weight together with the IV display. The customer orders an item from the VR store, and by cross-checking with the store, the corresponding weight of the item is unleashed using the motor gear combination on the user’s hand. The previous version of the device was planned and tested with the GSM technology; however, this version has various improvements and uses the VLC technology with an increased data transfer rate. Equalization technology has also been built to improve the signal coverage and aid the received data decoding process.

### 4.1. Pre-Equalization and Post-Equalizer

**Figure 6:**
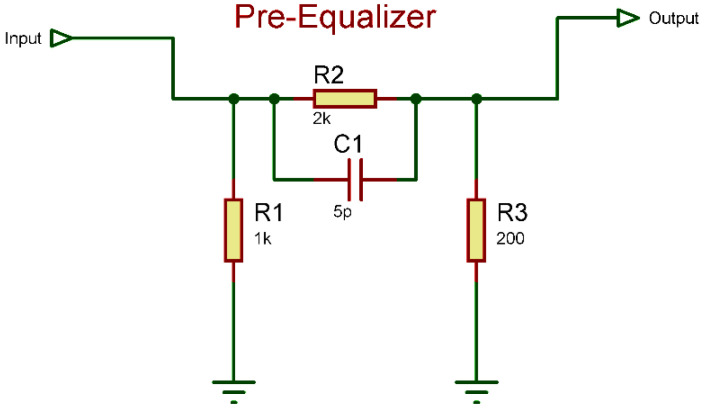
RC Pre-Equalizer circuit diagram (schematics).

Having a wide variety of frequency responses from different electronic components and considering the fact that LED has a limited bandwidth, a pre-equalizer circuit is required to increase the bandwidth of the commercial LED, which, at the cost of gain, increases the bandwidth and the gain reduced is increased by the amplifier stage configuration. For this project, an RC pre-equalizer circuit is used. The pre-equalizer frequency response is above 100 MHz (shown in Figure 6) but the amplifier stage used has a cutoff of around 10 MHz, thereby making the overall frequency response around 10 MHz.

The component values for the pre-equalization circuit are designed following the reversal of the measured frequency response. The circuit is a high-pass filter circuit with a designed attenuation of around 35dBm (~3.2 watts) and has a transfer function expression of:

$$H\left(\omega \right)=\frac{R_{3}}{R_{3}||X_{C_{1}}+R_{3}}=\frac{R_{3}}{\frac{R_{3}\times j\omega C_{1}}{R_{3}+j\omega C_{1}}+R_{3}}$$

**Figure 7:**
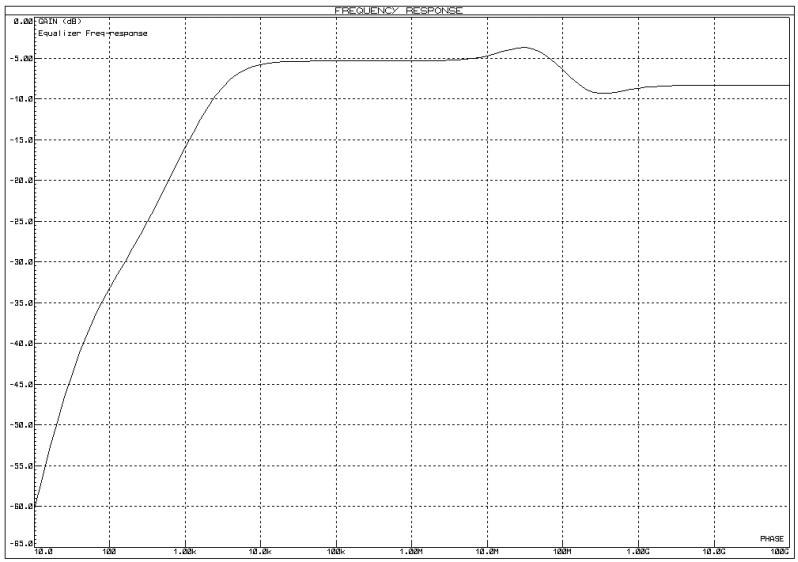
Frequency response of the RLC pre-equalizer in Figure 6.

One set of values that placates the working above 10 MHz frequency leads to results involving component values of $R_{1}=1000\;\Omega ,\;C_{1}=5\;pF,R_{2}=2\;k\Omega ,R_{3}=200\;\Omega .$ The frequency response of the circuit from Figure 6 is shown in Figure 7. In Figure 7, the y-axis represents the gain of the pre-equalizer circuit output in the dB scale while the *x*-axis corresponds to the frequency (in Hertz, Hz).

**Figure 8:**
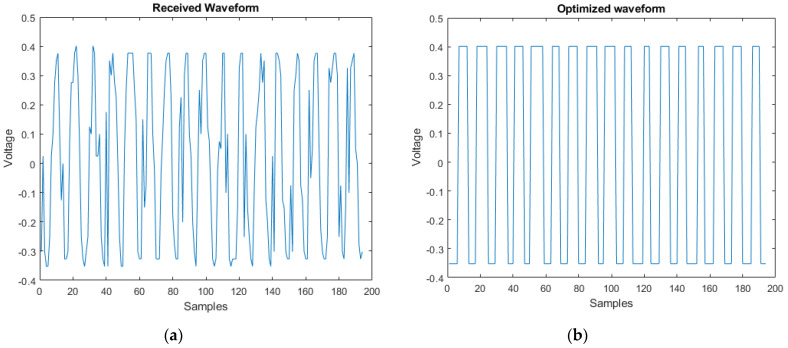
(**a**) The received signal (before DPE) and (**b**) after DPE.

For the post-equalization (to improve the frequency and amplitude of the received signal), DPE (firmware created on Arduino Nano) is castoff and henceforth, the signal before and after the process of the DPE for 200 samples is shown in Figure 8.

### 4.2. Device

**Figure 9:**
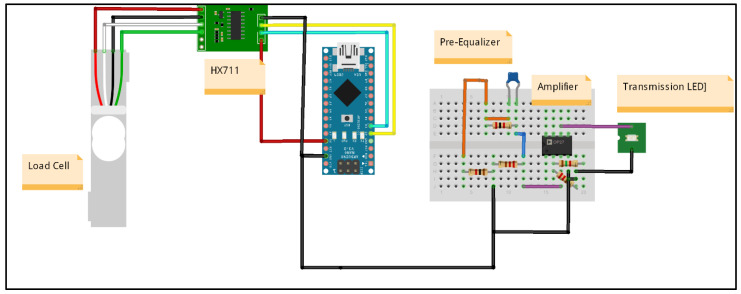
VLC tele-weight sending end schematic diagram.

**Figure 10:**
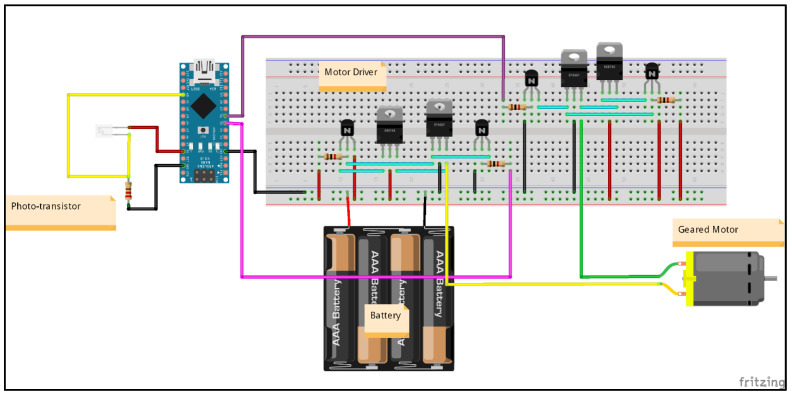
VLC tele-weight receiving end schematic diagram.

The sending end schematic diagram (shown in Figure 9) includes the configuration of the pre-equalizer (to increase bandwidth), amplifier (to increase the gain), and transmission LED to transmit the pulse signal corresponding to the input weight while the receiving end (shown in Figure 10) includes dual H-bridge driver connected with a motor to adjust the position based on the received input weight value. The motor driver circuit on the breadboard is a dual H-bridge driver (motor driver) that allows the motor to rotate clockwise as well as in an anti-clockwise direction and the surplus power required to operate is provided by the 5–9 V external battery.

### 4.3. Performance Evaluation

**Figure 11:**
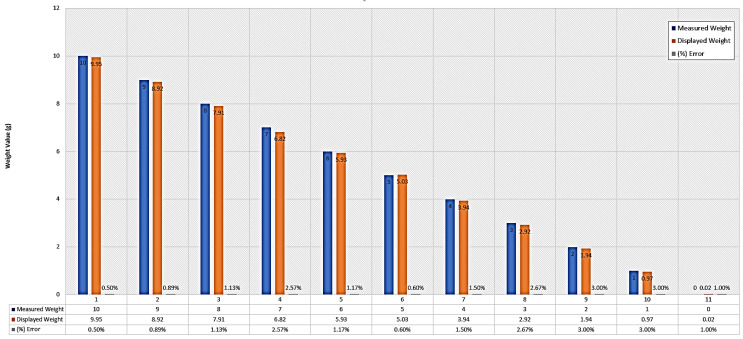
Results from the trial on VLC tele-weight device between 0 and 10 kg weight.

To determine the performance evaluation for the proposed system, repeated trials are performed and the histogram chat (containing percentage error, sent weight, and received weight) is shown in Figure 11. A maximum percentage error of 3% is achieved with the lowest error value at around 0.50%.

A few repeated trials are made for the weight in the range of 0 to 10 kg (by incrementing 1 kg in each trial) and from the obtained results, a confusion matrix based on predicted (P) and actual (A) values is generated. The cumulative weight sent in all trials is 55 kg while the received weight from all trials is 54.35 kg, thus generating a confusion matrix as:

$$Confusion\;matrix=\begin{array}{cc} \begin{array}{c} n=11\\ A:YES\\ A:NO \end{array} & \begin{array}{c} \begin{array}{cc} P:YES & P:NO \end{array}\\ \left[\begin{array}{cc} 54.35 & 0.65\\ 0.113 & 0.537 \end{array}\right] \end{array} \end{array}$$

A brief assessment involving the current system offering various improvements compared to the previous version (indicated in [3] that was created employing the global system for mobile communication (GSM)) is shown in Table 1.

## 5. Conclusions and Future Work

With the emerging evolution of the smart cities concept, different governments and research organizations are planning to include diverse frameworks involving smart embedded systems, 6G communication technologies, embedded IoT, and Li-Fi involvement in the process. The current problem with the virtual stores and HMD is that they allow the user to visualize the avatar model but there is not much haptic interaction with the product. This research work aims to revolutionize the virtual store shopping aspect by introducing the improved version of the tele-weight device that works with the 6G Li-Fi (VLC) technology and allows haptic interaction by introducing the weight-based sensation with the product.

The tele-weight device proposed in this research work has a three-fold contribution to be discussed. The first part is the sending end part which is connected with the end user store administration, while the second part is the VLC communication technology, and the third part is the receiving end part. For experimentation purposes, the sending part includes the weight measurement circuitry using Arduino Nano as an embedded micro-control unit, while in the receiving part, another set of the Arduino Nano together with the motor driver makes the motor rotate using an H-bridge driver. VLC communication technology consists of the pre-equalizer circuit connected with the LED, followed by an amplifier to improve the frequency transmission range and DPE at the receiver side to programmatically process the signal by acting as an equalizer filter. Various trials are performed to determine the accuracy and the percentage error in each trial ranges from 0.5 to 3%, accordingly.

With the focus of the industry being driven towards the concept of the metaverse and graphical computing, the funding partners are looking for haptic sensing technology to introduce immersive HMD headsets. The current form of the device has certain limitations (such as the non-linear behavior of the weight sensor, the need for a line of sight (LOS) link, and VLC limiting factors including beam dispersion, atmospheric absorption, shadowing, etc.) together with the absence of any commercially accessible VLC modules and Li-Fi modules, due to which fact, a circuit to increase the transmission LED bandwidth is created in this work. A future recommendation for the prototype is to implement it with the hybrid Li-Fi computing technology and reveal the full product over the headset together with the VR store.

## Figures and Tables

**Table 1:** Comparison between the current and the previously created system.

Parameter	Tele-Weight Device Using GSM.	VLC Haptic Tele-Weight Device
**Scope**	Works with designed accuracy and is slower in displaying weight.	Works by using the capability of light and is faster in performance.
**Transmission speed**	Up to 9.6 Kbps	Up to 10 MHz
**Supported weight**	0–5 kg	0–10 kg
**Transmission time (time factor)**	1.68–2 min	0.1–1 µs
**Communication technology**	Previous generation GSM	6G-based VLC transmission
**Requirement**	High level of training and expertise required to use this system	Medium expertise is required to use this system.
**Primary focus**	Smart city	Communication technology such as 6G upgrades

## Data Availability

No dataset is used. Experiment is performed on the prototype.
